# A Data-Based Approach to Discovering Multi-Topic Influential Leaders

**DOI:** 10.1371/journal.pone.0158855

**Published:** 2016-07-14

**Authors:** Xing Tang, Qiguang Miao, Shangshang Yu, Yining Quan

**Affiliations:** School of Computer Science and Technology, Xidian University, Xi’an, China; Beihang University, CHINA

## Abstract

Recently, increasing numbers of users have adopted microblogging services as their main information source. However, most of them find themselves drowning in the millions of posts produced by other users every day. To cope with this, identifying a set of the most influential people is paramount. Moreover, finding a set of related influential users to expand the coverage of one particular topic is required in real world scenarios. Most of the existing algorithms in this area focus on topology-related methods such as PageRank. These methods mine link structures to find the expected influential rank of users. However, because they ignore the interaction data, these methods turn out to be less effective in social networks. In reality, a variety of topics exist within the information diffusing through the network. Because they have different interests, users play different roles in the diffusion of information related to different topics. As a result, distinguishing influential leaders according to different topics is also worthy of research. In this paper, we propose a multi-topic influence diffusion model (**MTID**) based on traces acquired from historic information. We decompose the influential scores of users into two parts: the direct influence determined by information propagation along the link structure and indirect influence that extends beyond the restrictions of direct follower relationships. To model the network from a multi-topical viewpoint, we introduce topic pools, each of which represents a particular topic information source. Then, we extract the topic distributions from the traces of tweets, determining the influence propagation probability and content generation probability. In the network, we adopt multiple ground nodes representing topic pools to connect every user through bidirectional links. Based on this multi-topical view of the network, we further introduce the topic-dependent rank (**TD-Rank**) algorithm to identify the multi-topic influential users. Our algorithm not only effectively overcomes the shortages of PageRank but also effectively produces a measure of topic-related rank. Extensive experiments on a Weibo dataset show that our model is both effective and robust.

## Introduction

Online social networks provide a medium through which millions of users interact with each other; their members diffuse information and exhibit influence [1]. Influence analysis has received wide research attention. Among the investigated problems, finding influential individuals is an important topic for many applications such as online advertising, recommender systems and information diffusion. Consider online advertising as an example. When a new movie or product is released, the producer wants the item to be discussed frequently on social networks. The producer selects a small number of initial users from the network to “retweet” it (an action in the twitter-like social network that enables users to disseminate a certain item). These retweets are expected to launch gain a large amount of audience attention. The problem lies in determining who should be selected as the initial users to gain the most influence within the social network. Outside of advertising, when some emergency news needs to be announced about a particular subject, it is essential to select the most influential users as the seed users who will spread that news effectively. Both of these problems are related to social influence ranking, a problem that has attracted many studies.

Twitter-like social networks employ a network model called “following”, in which each user is allowed to follow anyone without requiring permission. Based on these established social relations, a user will be alerted whenever a user they are following posts tweet updates [2]. Hence, this structure is the major means of influence propagation in these networks. Moreover, algorithms akin to PageRank [3] [4] have been used to find influential users on social networks whose topology is similar to the web. In PageRank, a random surfer is assumed to browse along links between web pages. However, most tweets are open-access for everyone in a Twitter-like social network, which means that users are able to retweet whatever they are interested in without the permission of the original poster. This open access breaks the restrictions of fixed social network structures. As a result, information freely propagates between users who may not necessarily have direct links in the social network. Most of the studies on social influence [5] [6] have targeted the actual retweet network instead of the “following” network. But topology is not the only means of information diffusion in social networks as it is in web networks. This paper decomposes the influential effects of a particular user into two parts: direct influence, which is triggered by a retweet action, and indirect influence, which is caused by the actions of others, without necessarily involving a following relationship.

In total, users generate an enormous number of tweets everyday on social media. Topic-mixed tweets propagate through the same network, and these various tweets contain the topic-related information. Faced with this flood of information, information seekers [7] are eager to follow only the most valuable users for a given topic. Actually, because sharing the same network structure, it is hard to distinguish different topics from the same information propagation. Discovering topic-dependent influential leaders enables user find the topic-related posters. On the other hand, Social network sites show new users a list of topics from which they can choose the ones they are most interested in following. Then, the most influential user for this topic will be recommended to the new user. Therefore, this “cold start” problem [8] could be well solved by recommending topic-related influential users. Importantly, influential users for one topic may fail to have the same influence for other topics, which means users play different roles in different topics [9]. Therefore, influence is topic-dependent, making it necessary to discover the topic-dependent influential leaders.

In this paper, we propose a novel influential model called the multi-topic influence diffusion (**MTID**) model to discover topic-related influential leaders. Specifically, the influence of these users in our model consists of both direct and indirect influence. The direct influence follows the information propagation trace along the links. Meanwhile, users can retweet tweets posted by people they are not following, giving the original posters further indirect influence. Notice that both types of influence are related to different topics. Based on **MTID**, we further propose a topic-dependent rank algorithm, namely **TD-Rank**. Different from setting one ground node to make the network strongly connected, as in LeaderRank [10] [11], we treat ground nodes as the different topic pools of original tweets to construct a topical view of the whole network. Connecting all the nodes with each ground node hence represents a one-topic view of the network. This view is strongly connected and, thus, has the same properties as LeaderRank. Moreover, the transition probability in our model is extracted from traces of tweet action including both posting of the original tweet and retweeting others’ tweets. This approach has been shown to be helpful in finding influential users [12]. We further experimentally demonstrate that our proposed ranking algorithm extracts nontrivial nodes as influential nodes in various topics on the large-scale Weibo network.

## Related work

Generally, discovering influential leaders is related to two research topics. The first is measuring the maximization of influence. Influence maximization aims to find a set of seed users that influence a large number of other users. This problem was first studied by Domingos and Richardson from an algorithmic perspective [13]. Then, Kempe et al. [1] formulated the problem as a combinatorial discrete optimization problem; they proved that this problem was NP-hard and proposed a greedy algorithm to deal with it. Based on this basic discussion, many recent studies have also focused on topic-dependent issues. Chen et al. [14] studied topic-aware influence maximization; they proposed an algorithm to find *k* seeds from social network such that the topic-aware influence was maximized. Bakshy et al. [15] investigated the diffusion cascades generated by 1.6M Twitter users using a follower graph. They found that the number of followers was an important indicator of an influential user, further evidence that structure is important. Cha et al. [2] used a large amount of data collected from Twitter and presented an in-depth comparison of three measures of influence: indegree, retweet and mentions. They made observations that most influential users can hold significant influence over a variety of topics, which also revealed the need to distinguish topic-dependent influence.

The second topic is the detection of authority nodes. One method for doing this is to create a rank list. Akin to the structure of the web, PageRank [3] [4] and the HITS algorithm [16] are also borrowed to investigate the influence-ranking problem. As a variant of PageRank, Lu et al. [10] proposed the LeaderRank algorithm to identify influential nodes by placing the ground node. Li et al. [11] further extended the algorithm by assigning degree-dependent weights to links associated with the ground node. All these methods determine influence based on a graph structure. By introducing the information propagation model [17], Zhu et al. [18] proposed a novel information diffusion model and integrated a Markov Chain into the independent cascade model. Based on this proposed model, they further proposed the rank algorithm SpreadRank to find influential users. To combine both greedy and heuristic algorithms, Cheng et al. [19] proposed IMRank, which found a self-consistent ranking by considering ranking-based marginal influence spread according to current ranking. TwitterRank [20] first measured the influence by taking both the topical similarity between users and the link structure into account. However, TwitterRank fails to distinguish the different types of topic-related influential leaders by assuming that tweets are retweeted according to a certain similarity. Some methods have tried to solve this problem with learning-based models. Su et al. [21] discussed the diversified expert-finding problem in academic social networks and proposed a new objective function to diversify the ranking list for a particular topic. Wang et al. [5] first introduced multi-task learning to predict individual influence based on the traces of information propagation.

Our work is different from all the studies described above in that we propose a novel influence diffusion model, to which we further add a novel topic-dependent rank algorithm. We introduce several ground nodes to decompose the total influence into direct influence and indirect influence. The adoption of ground nodes allows the construction of multiple-topic views of the whole network. Moreover, this is helpful in finding topic-related influence. Meanwhile, it also overcomes the shortages of PageRank. In addition, we adopt a data-based approach to define the transition probability, which makes the model more accurate.

## Materials and Methods

### Multi-topic influence diffusion model

First, a directed social network *G* = {*V*, *E*} is formed consisting of users and their following relationships, where *V* is the set of users and *E* represents the edge set of their followings. We denote *e* = <*i*, *j*> in *E* as a directed edge starting from *i* to *j*. Meanwhile users have traces of tweets *D* = {*d*_1_, *d*_2_, …, *d*_*N*_}, where *d*_*i*_ is trace of user *i*, and *N* is the number of users. We separate these tweets into two types: original tweets, those that are created originally by that user, and retweets, which are re-posted tweets created by others.

Based on the collected tweets, we conduct topic distillation, which aims to automatically identify the topics that users are interested in. For this purpose, the Latent Dirichlet Allocation (**LDA**) model [22] is applied. As a result, we denote $P_{i}^{o}$ as the topic distribution of original tweets posted by user *i*, while $P_{i}^{r}$ is user *i*’s retweet topic distribution.

Using the above measurements, we describe the multi-topic influence diffusion model (**MTID**) in greater detail. First, suppose user *j* retweets one tweet from user *i*. Then, user *i*’s influence is expanded by user *j* through user *j*’s influence. We then set *I*(*i*) as the influence of user *i*. As stated above, we decompose the total influence of particular user as follows:

$$\begin{array}{c} I\left(i\right)=I_{d}\left(i\right)+I_{r}\left(i\right),\;i=1,2,...,N \end{array}$$ (1)

where *N* is the number of users, *I*_*d*_(*i*) is the direct influence gained from followers, and *I*_*r*_(*i*) is the indirect influence caused by retweets from users who are not followers of user *i*. Moreover, taking the topic factor into consideration, we define the influence of topic *t* as *I*^*t*^(*i*). Thus, Eq (1) can be rewritten as:

$$\begin{array}{c} I^{t}\left(i\right)=I_{d}^{t}\left(i\right)+I_{r}^{t}\left(i\right) \end{array}$$ (2)

Before proceeding to deal with the definition of $I_{d}^{t}\left(i\right)$ and $I_{r}^{t}\left(i\right)$, we make two observations about Twitter-like social networks:

*Observation 1*: A user’s posted tweets may be accessed by anyone in the whole social network due to the policies of online social network sites.

*Observation 2*: A follower’s retweet action is the primary way by which users enlarge their scope of influence, which means that their influence diffuses as the tweet propagates along the network. More influential users will gain more retweets.

**Fig 1:**
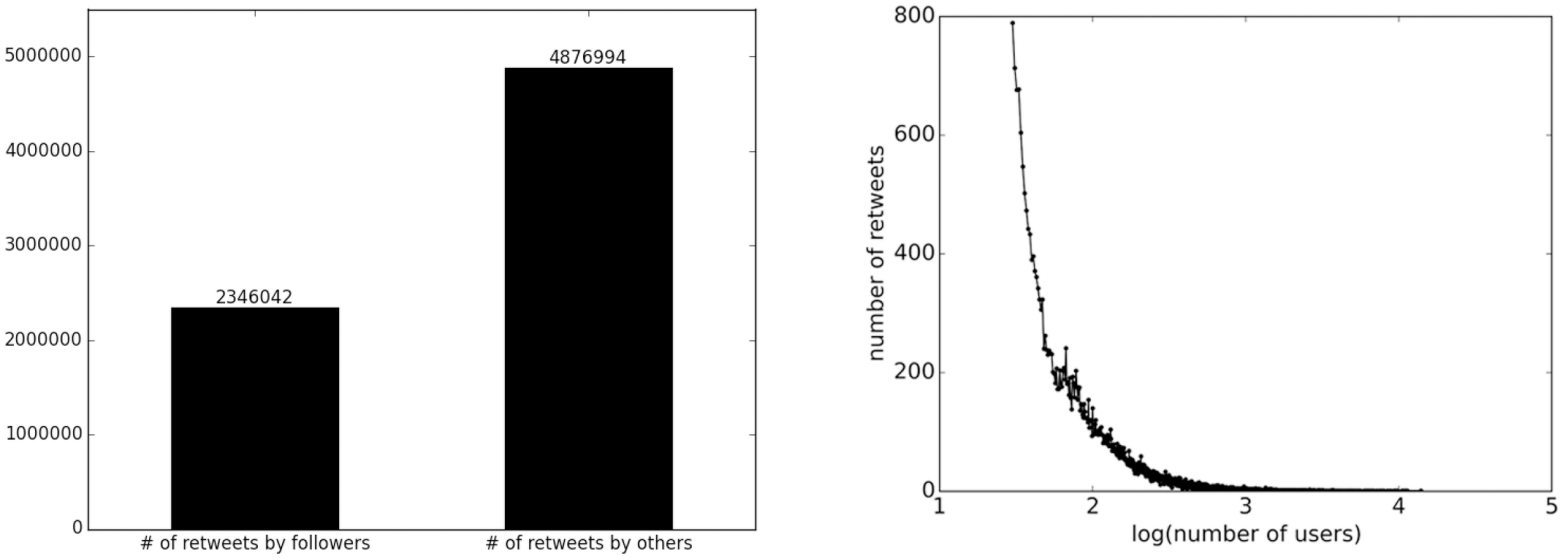
The statistics of Weibo data. (a) the retweet source distribution, (b) the number of retweets distributed over log(number of users).

To verify these two observations, we calculated the statistics on our crawled data in Result Section, as illustrated in Fig 1.

In Fig 1(a), we count how many retweets are retweeted from followers and from other users who are not direct followers. Obviously, users can not only retweet tweets from people they are following but also those that originate from people they are not following. This statistic can validate *Observation 1*, and also clarifies the distinction between direct influence and indirect influence. We illustrate the distribution of retweet numbers per user in Fig 1(b). To show the distribution clearly, we take the log value of the number of users. As shown, most tweets are retweeted by fewer than 100 users. Only a few users have a larger retweet range, indicating that only the most influential leaders can gain large retweet numbers. This can be the consequence of *Observation 2*.

To explain *Observation 1*, we introduce *topic pools*. Topic pools are public information sources from which any user is able to find and retweet original tweets. This can be interpreted as a function of “*discovery*” (http://d.weibo.com/) in Twitter-like social network sites. The discovery function aims to enable users to find topic-related tweets despite the lack of existing following relationships.

Assume user *i* has a topic distribution $P_{i}^{o}$ on his original tweets. The component of topic *t* is $p_{\left(i,t\right)}^{o}$. When user *i* posts an original tweet, that tweet contributes to the topic *t* pool with an expected value of $p_{\left(i,t\right)}^{o}I^{t}\left(i\right)$. As a result, the influence of each topic pool *I*^*t*^(*g*) consists of the combination of topic contributions from all the users:

$$\begin{array}{c} I^{t}\left(g\right)=\sum_{i=1}^{N}p_{\left(i,t\right)}^{o}I^{t}\left(i\right) \end{array}$$ (3)

According to Eq (3), a user with higher influence will contribute more to the topic pool, indicating that more credits will be obtained if one tweet is retweeted by this user. Every topic pool is equally likely to distribute its influence among the whole network. Users are expected to gain $\frac{1}{N}I^{t}\left(g\right)$ influence from the topic pool. Therefore, the influence contributed by each user will be distributed among whole network via topic pools. Thus, we define the indirect influence of user *i* as:

$$\begin{array}{c} I_{r}^{t}\left(i\right)=\frac{1}{N}I^{t}\left(g\right) \end{array}$$ (4)

According to Eq (4), user *i* will gain influence indirectly through others without gaining any new follower relationships. Specifically, the influence of user *i* can expand rapidly when users with higher influence perform retweets.

Direct influence is gained from *i*’s followers immediately. Suppose *F*_*i*_ = {*f*_1_, …, *f*_*m*_} denotes the follower list of user *i*, and *m* is the number of followers. Then, the direct influence can be defined as follows:

$$\begin{array}{c} I_{d}^{t}\left(i\right)=\sum_{j\in F_{i}}p_{\left(i,j,t\right)}^{r}I^{t}\left(j\right) \end{array}$$ (5)

where $p_{\left(i,j,t\right)}^{r}$ is the retweet topic distribution between user *i* and user *j* on topic *t*. The Eq (5) explains the *Observation 2*. When user *j* retweets user *i*’s tweets, the influence transits from *j* to *i*. Conversely, if *j* does not retweet any of *i*’s tweets, the $p_{\left(i,j,t\right)}^{r}$ will be 0, meaning that user *j* only reads *i*’s tweets but never retweets them.

Our **MTID** model is able to explain both the observations well from a data viewpoint. Note that our proposed model is different from the random surfer model in other algorithms; instead, our model is in accord with the available Tweet data and is not susceptible to the effects of manually determined parameters, which has further effects in the ranking algorithm based on the **MTID** model.

### Topic Dependent Rank algorithm

**Fig 2:**
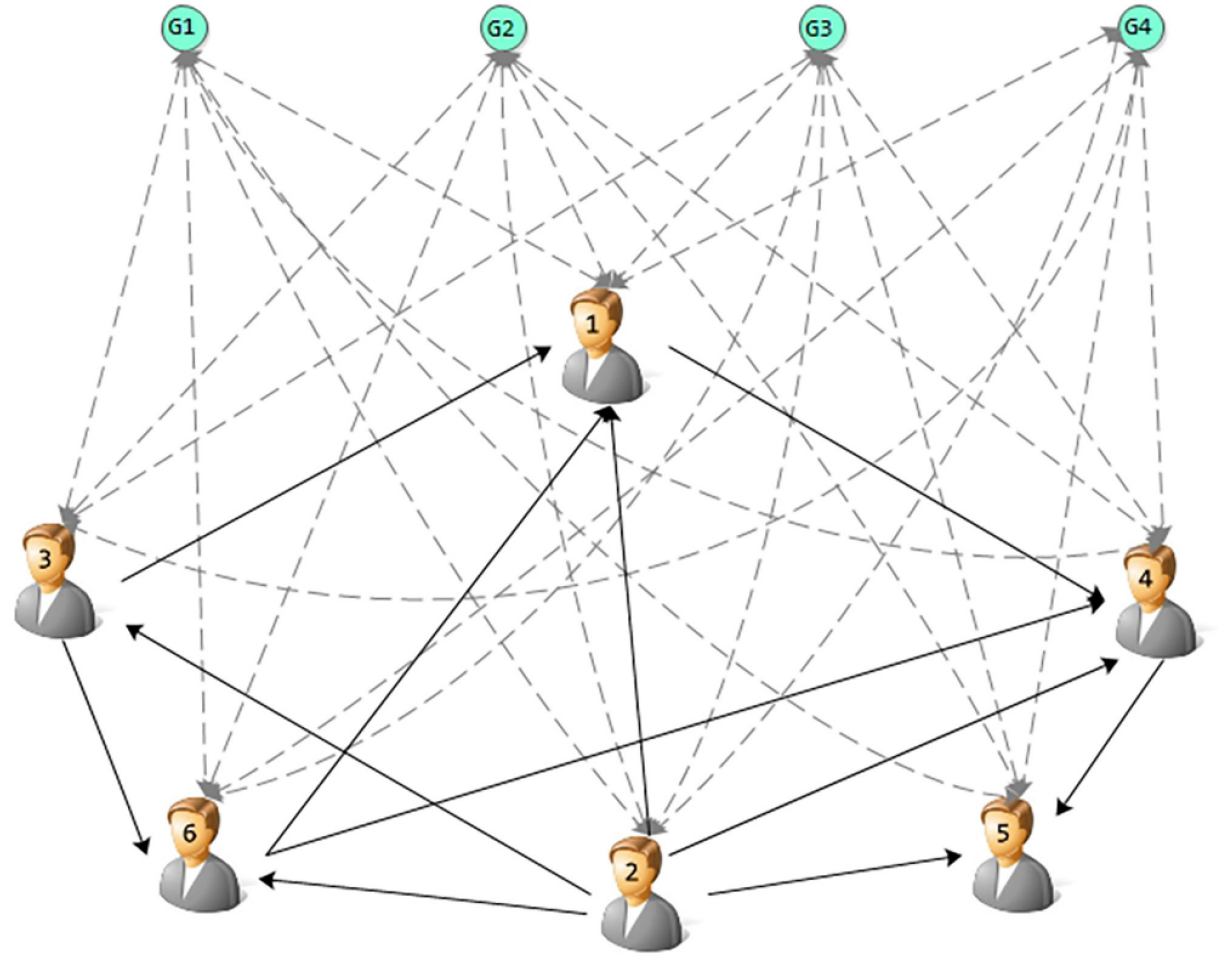
An illustration of the ground nodes. There are four ground nodes in this network representing four topics.

Inspired by LeaderRank, we model topic pools as ground nodes inserted into the network as illustrated in Fig 2.

In the Fig 2, the ground nodes will establish bidirectional relations with each node. The network thus becomes strongly connected and consists of *N* + *T* nodes and *M* + T*N links, where *N* is the number of nodes, i.e users, *M* is the number of edges, and *T* is the number of topics. In addition, the weight is set as $p_{\left(i,t\right)}^{o}$ for the inbound direction from user *i* to ground node *g* representing topic *t*, while the outbound direction is $\frac{1}{N}I^{t}\left(g\right)$.

Therefore, we show the update rules for users’ scores defined as $s_{i}=\left\{s_{i}^{1},...,s_{i}^{T}\right\}$ and the ground node scores on topic *t* as $s_{g}^{t}$, respectively, as follows:

$$\begin{array}{c} s_{g}^{t}=\sum_{i=1}^{N}p_{\left(i,t\right)}^{o}s_{i}^{t} \end{array}$$ (6)

where $s_{i}^{t}$ is the score of user *i* on the topic *t*. Moreover, we have

$$\begin{array}{c} s_{i}^{t}=\sum_{j\in F_{i}}p_{\left(i,j,t\right)}^{r}s_{j}+\frac{1}{N}s_{g}^{t} \end{array}$$ (7)

Notice that methods for calculating influential score by Eqs (6) and (7) are in accordance with the **MTID** model. In addition, we define the final score of a user to be the topic-dependent leadership score vector **S** including scores on all topics, namely,

$$\begin{array}{c} S_{i}=s_{i}\left(Iter_{c}\right)+\frac{s_{g}\left(Iter_{c}\right)}{N} \end{array}$$ (8)

where **s**_**g**_(*Iter*_*c*_) is the vector expression of ground node scores on all of the topics at steady state, and **s**_**i**_(*Iter*_*c*_) is the vector expression of user *i*. The TD-Rank algorithm is summarized in Algorithm 1.

**Algorithm 1** TD-Rank algorithm

**Input**: Network *G* = {*V*, *E*}, tweets associated with every user *D* = {*D*_1_, …, *D*_*N*_}, topic number *T*; the error threshold *ϵ* to stop the iteration; the maximum iteration times *Iter*_*max*_

**Output**: TD-Rank score of list *TDS* = [**S**_**1**_, …, **S**_**N**_]

 process *D* with LDA according to the topic number *T*

 **for** *t* = 1 TO *T* **do**

  $s_{g}^{t}=0$

 **end for**

 connect ground nodes to users with bidirectional edges

 set the weights on edges

 **for** *i* = 1 TO *N* **do**

  *TDS*[*i*] = **S**_**i**_ = [1/*N*, …, 1/*N*]

 **end for**

 **while** *err* > *ϵ* or *k* < *Iter*_*max*_ **do**

  *Temp* = *TDS*_*k*_

  Update every ground node score according to Eq (6)

  Update every user score according to Eq (7)

  find the max error: *err* = *Temp* − *TDS*_*k*_

 **end while**

In other algorithms akin to Pagerank, the final ranking equation can be defined as:

$$\begin{array}{c} PR\left(i\right)=\alpha +\left(1-\alpha \right)\sum_{<i,j>\in E}p_{j,i}PR\left(j\right) \end{array}$$ (9)

where *PR*(*i*) is the rank value of node *i*, *α* is the decay factor (i.e. the return probability), and *p*_*j*,*i*_ is the transition probability matrix defined by the specific algorithm.

There are several drawbacks in applying this algorithm to social networks. First, the return probability is essential. Convergence is guaranteed only on strongly connected networks. In addition, the probability on every edge is identical for all users—irrespective of each user’s tweet history. In comparison, our proposed TD-Rank based on the **MTID** effectively overcomes these shortages. Due to the adoption of ground nodes, TD-Rank extends the advantage of LeaderRank on every topic view. Moreover, we further adopt a data-driven approach to divide the transition probability into original and retweet probability. Finally, we reconstruct the network into a strongly connected network using the ground nodes and adopt a data-based approach to deal with the transition probability between users and ground nodes, aiming to discover an actual influence score.

## Results

### Datasets and experiment settings

To validate the effectiveness of the TD-Rank algorithm, we test it on crawled data from Weibo, the largest twitter-like social network in China. We start by randomly choosing several active seed users to avoid “Zombie users”—those who have registered but have not posted any tweets. Specifically, we include active users who retweeted more than 20 tweets between May 24th 2013 and May 24th, 2014. With these users, we crawl a network with 211,000 users, 1,612,289 following relationships and 47,002,906 total tweets. The detaileded statistics for this dataset are are listed in Table 1.

**Table 1:** Dataset Statistics.

Users	Retweets	Original tweets	Relations
211,000	7,223,036	39,779,870	1,612,289

The only parameters that must be set are the LDA parameters, which reflect the number of ground nodes selected. The LDA is tuned by three parameters: the Dirichlet hyper-parameters *α*, *β* and topic number *T*. In this paper, these parameters are set as *T* = 20, *α* = 50/*T* + 1, and *β* = 0.1 + 1 in Spark [23]. Obviously, choosing different values for these parameters has implications for the model results. However, this is a basically a model selection problem, which is not the focus of this paper. Then, we conduct LDA on traces of tweets *D* for every user. Table 2 lists 5 topics obtained by LDA as an example and the top 5 associated translated words in each topic.

**Table 2:** Examples of topics and associated words extracted by LDA.

Topic #	1	2	3	4	5
Associated words	company	user	children	designs	match
	management	technology	parents	photo works	sports
	marketing	intelligence	education	photography	the world cup
	brand	APPs	teacher	creativity	seasons
	market	Android	cultivation	style	NBA

### Experiment results

In this section, we make comparisons against related algorithms using the above dataset with Spark. The related algorithms studied include:

- **PageRank**, which measures the influence by taking the link structure of the network into account. The experiment setting in the comparison is set as follows: the return probability is 0.15 and the transition probability is $p_{ij}=1/k_{i}^{out}$, representing the probability that *i* goes to *j*, while $k_{i}^{out}$ denotes the outbound degree of *i*.
- **LeaderRank**, which introduced a ground node to connect all of the nodes, setting the transition probability as $p_{ij}=1/k_{i}^{out}$.
- **TwitterRank**, which first studied topic-related ranking. In this comparison, we extract the same topics using LDA in TD-Rank.

The first experiment discusses the topic property of the rank result. We introduce the entropy on topic distribution. The entropy is calculated by:

$$\begin{array}{c} E_{i}=-\sum_{t=1}^{T}p_{i,t}^{o}\log\left(p_{i,t}^{o}\right) \end{array}$$ (10)

**Fig 3:**
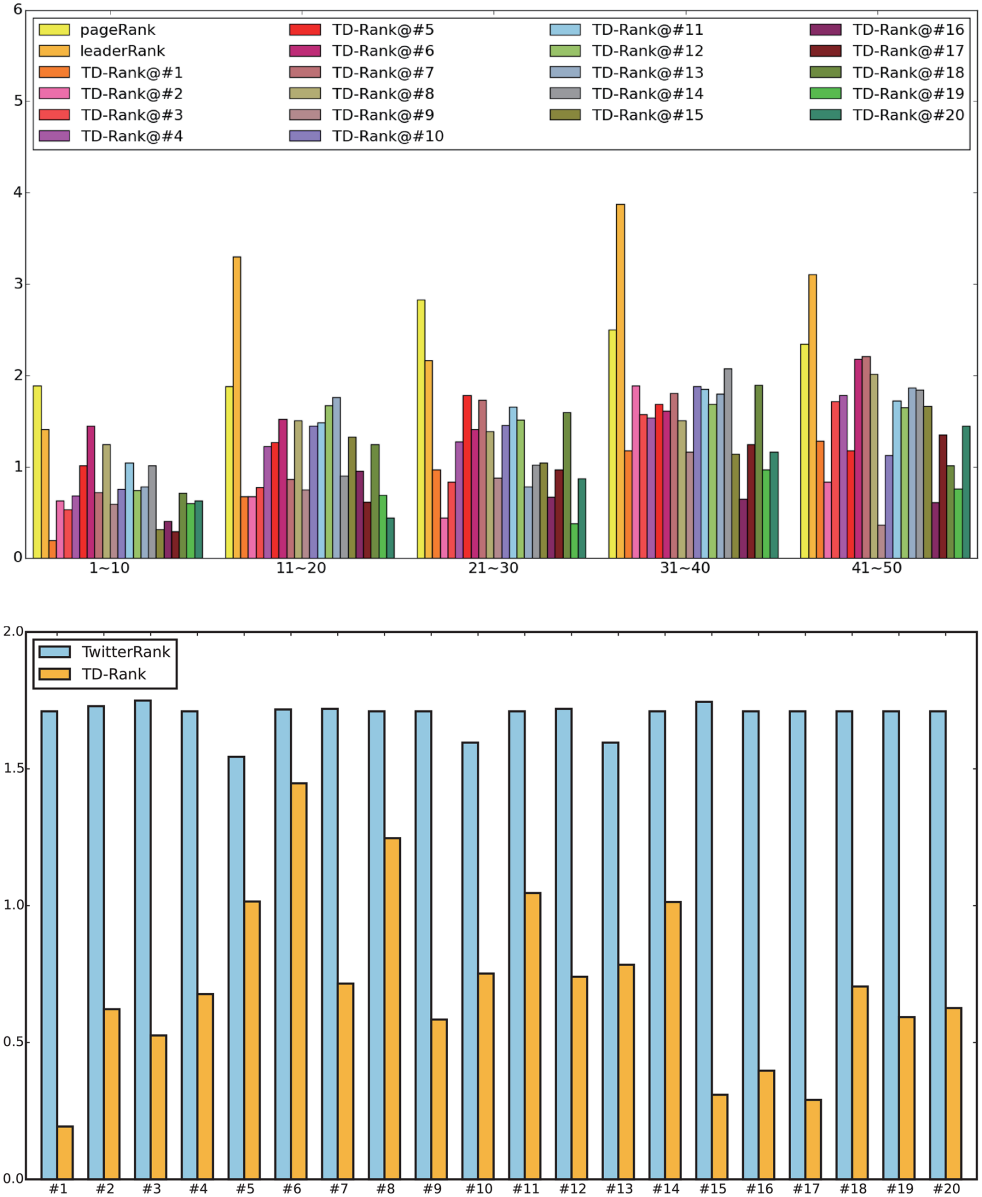
The topic entropy. (a) Comparison between PageRank, LeaderRank and TD-Rank; (b) Comparison between TD-Rank and TwitterRank on top 10 users.

Notice that we use the topic distribution on the original tweets here because original tweets are posted by a user to express his or her own interests. Then, we obtain the average entropy of users grouped in ranking list order as 1-10, 11-20, 21-30, 31-40 and 41-50. The comparison results are demonstrated in Fig 3. For ease of visualization, we first compare PageRank, LeaderRank and 20 topic-related TD-Rank results in Fig 3(a). For TwitterRank, we select top 10 users in every topic as an example and compare the results with TD-Rank in Fig 3(b).

From the results, it is obvious that the users ranked by TD-Rank and TwitterRank have far less entropy compared with PageRank and LeaderRank, indicating that our proposed algorithm finds topic-related influential leaders similarly to TwitterRank. Moreover, in Fig 3(b) the ranking of users by TwitterRank has more entropy than the ranking by TD-Rank, indicating that users in our ranking list are more closely related to the same topic.

Another issue concerning the ranking results is the problem of robustness. Many spammers exist in social networks who attempt to gain reputation for advertising purposes [24]. To investigate this issue, we create the *v* edges which link *v* fake followers to every user and observe the positional changes in the ranking.

**Fig 4:**
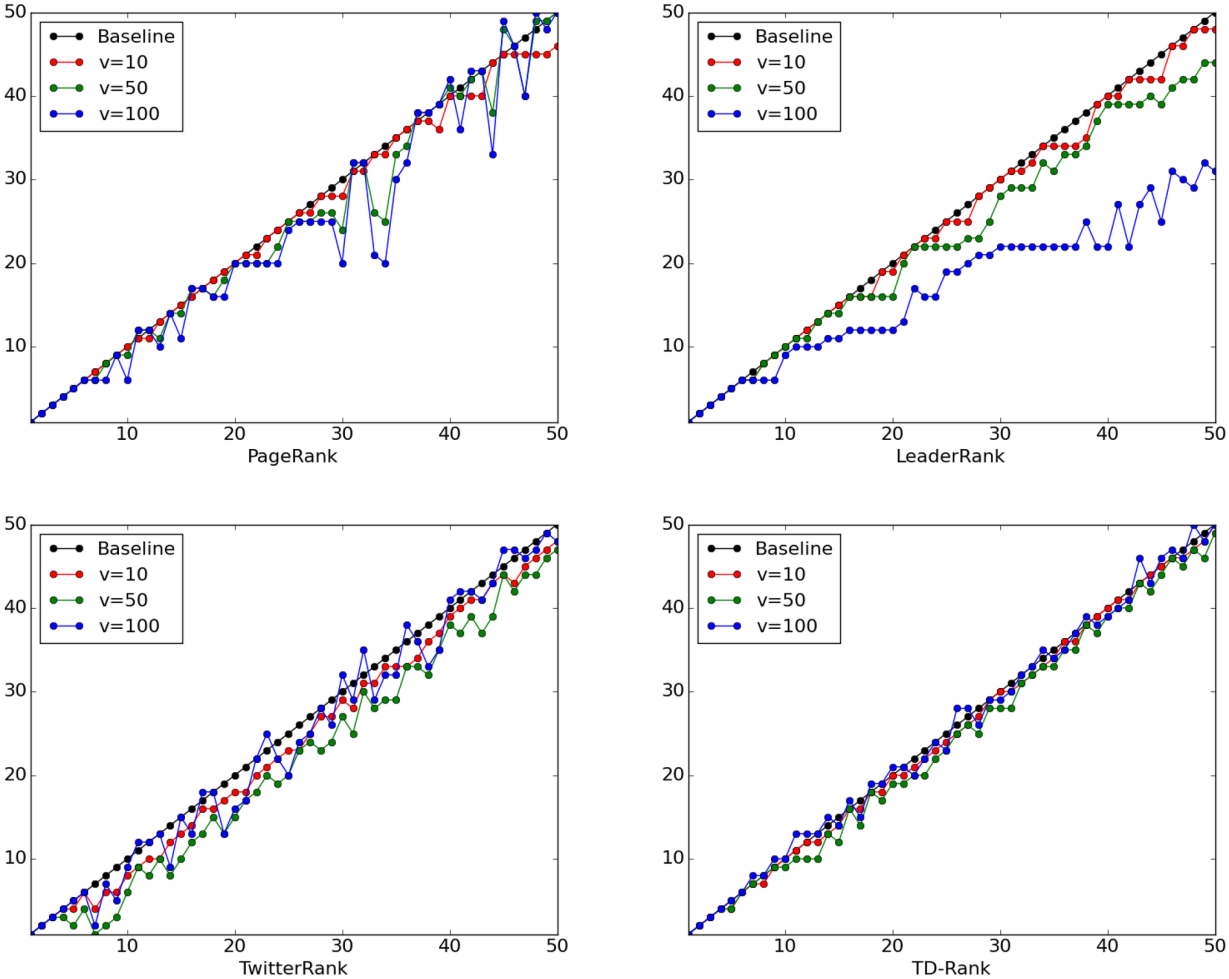
The spammer effect on ranking results. (a) PageRank, (b) LeaderRank (c) TwitterRank, (d) TD-Rank.

Specifically, we simulate the situation where a user creates *v* fake spammers and compare the positional changes in both ranking results. The whole process is described as follows. Suppose the user is *i*, we randomly select *v* users denoting as {*u*_1_, *u*_2_, …, *u*_*v*_}. Then following directed links are created to disturb the algorithm: { < *u*_1_, *i* >, < *u*_2_, *i* >, …, < *u*_*v*_, *i* >}. The results are reported in Fig 4.

The horizontal axis of Fig 4 shows the original rank of a particular user, and the vertical axis is the manipulated rank after the addition of *v* spammers. As result, the diagonal line is the baseline, which indicates the rank is invariant. Vertical shift from the diagonal line corresponds to the change of ranking results. In addition, for ease of visualization, we select the ranking list on topic #1 as an example to show the comparative performance of TwitterRank and TD-Rank. As shown, TD-Rank is the most robust against spammers because the changes in ranking positions are much smaller than those of the other algorithms, and TwitterRank is more robust than PageRank and LeaderRank. Therefore, the results are mainly due to the distinguishing topics. In all, TD-Rank is a better algorithm for creating robust rankings in social network.

The next issue is influence maximization. We first consider two information-diffusing models in previous work [25].

**Independent Cascade (IC) Model**: This model begins with an initial set of active nodes. The process unfolds in discrete steps. When node *v* first becomes active in step *t*, it has a single chance to activate each currently inactive neighbor *w* based on parameter *p*_*v*,*w*_. In our experiment, we set the parameter *p*_*v*,*w*_ uniformly to 0.1.

**Topic Independent Cascade (TIC) Model**: This model uses the same process as IC, but its the parameter *p*_*v*,*w*_ is related to topic. Specifically, the probability of diffusion is defined as:

$$\begin{array}{c} p_{v,w}=\sum_{t=1}^{T}\gamma_{v}^{t}p_{v,w}^{t} \end{array}$$ (11)

where $\gamma_{v}^{t}$ is the topic probability of user *v* on topic *t*, and $p_{v,w}^{t}$ represents the influence strength exerted by user *v* on *w* on topic *t*.

**Fig 5:**
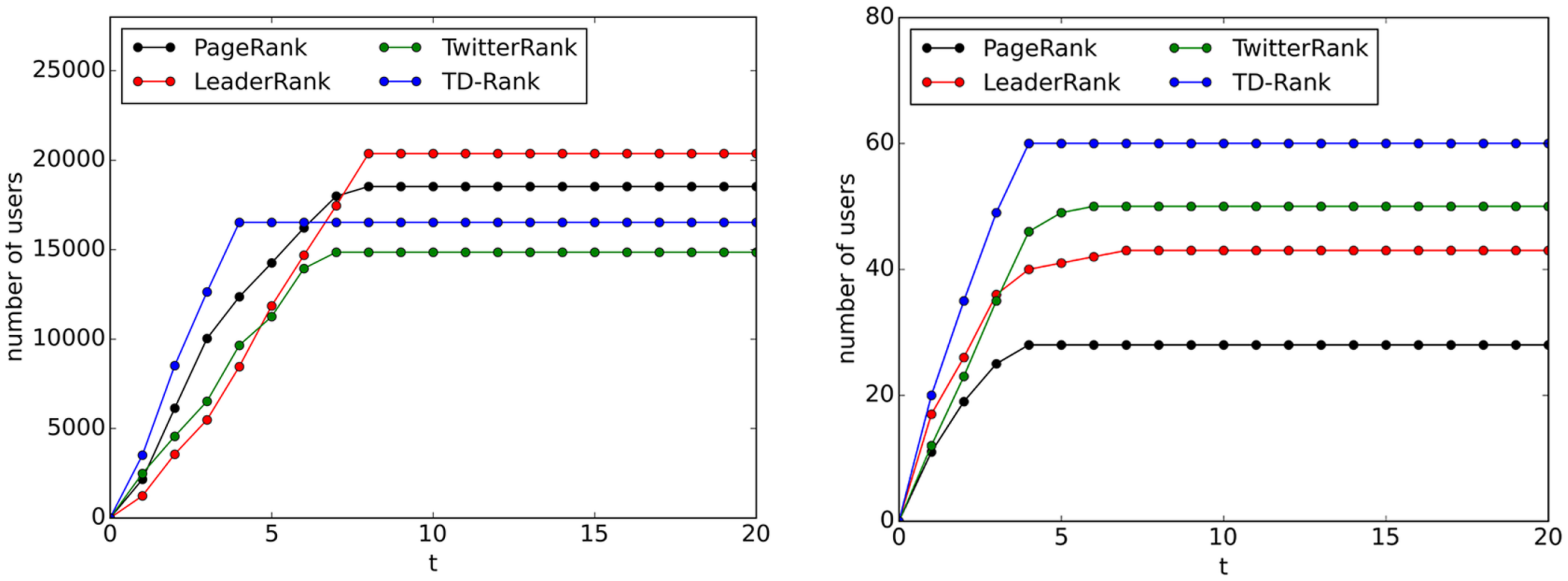
Comparison of influence maximization. (a) The results of IC Model. (b) The results of TIC Model.

We conduct experiments on IC and TIC with the top 10 users in every ranking results consisting of the initial set of active nodes. In addition, the top 10 users in TD-Rank and TwitterRank are also selected from the ranking list of topic #1 as additional experiments. The results are shown in Fig 5.

Because it ignores the effects of topic distribution, the IC model is more suitable for a comprehensive ranking algorithm. However, it is worth noting that TD-Rank converges to a stable number faster than the others, which indicates that TD-Rank is more inclined to measure the influence of users on one particular topic with a smaller population. Compared to TIC, TD-Rank and TwitterRank calculate the influence on more users by considering topic effects. In all, this experiment shows the essential value of taking the topic factor into account.

We also extracted the number of retweets for several tweets posted by the top 50 users in the whole dataset. Then, we investigated the differences between this true retweet number and the number obtained by TIC. Assuming that the set of tweets posted by user *i* is denoted by *d*_*i*_, we then evaluate the influence-predicting performances of all the algorithms using the mean squared error (MSE) as the evaluation metric:

$$\begin{array}{c} MSE=\frac{1}{N}\sum_{i=1}^{N}(\frac{1}{K}\left(\sum_{k\in d_{i}}{\left({\hat{V}}_{i}\left(k\right)-V_{i}\left(k\right)\right)}^{2}\right) \end{array}$$ (12)

where ${\hat{V}}_{i}\left(k\right)$ is the predicted number of retweets of a tweet *k* posted by user *i*, *N* is the number of users, and *V*_*i*_(*k*) is the real value.

The predictions’ MSEs are presented in Table 3. Note that the settings for TwitterRank and TD-Rank are the same as in the other experiments.

**Table 3:** Comparison of the predictions’ MSEs.

	PageRank	LeaderRank	TwitterRank	TD-Rank
MSE	29.95	27.21	18.03	16.38

As shown in Table 3, PageRank and LeaderRank perform the worst, followed by TwitterRank. TD-Rank performs the best, outperforming the competition in terms of MSE, which indicates that the influential leaders discovered by our proposed algorithm are closer to the true situation.

## Conclusion

In this paper, we focused on discovering multi-topic influential leaders in social network. We proposed a multi-topic influence diffusion (**MTID**) model, which decomposes the influence of a particular user into two parts: direct influence, which is influence related to that user’s followers, and indirect influence, which is influence that is not restricted to direct followers. To cope with the definition of indirect influence, we introduced topic ground nodes that represent topic pools for establishing links between users. Moreover, to deal with the transition probability, we adopted a data-based approach that extracts the topic distribution from traces of tweets. Based on **MTID**, we further proposed a topic-dependent rank (**TD-Rank**) algorithm to identify the topic aware influential leaders. Finally, we conducted extensive experiments comparing the existing ranking algorithms using the Spark platform. The experimental results demonstrated that our proposed algorithm is more robust, more accurate and more sensitive to topic than previous algorithms.

Our plans for future work include dealing with the dynamic structure of a following social network by incorporating a time factor into our model. We will also consider other influence measures in the future.
